# Converse theorems and the local Langlands correspondence in families

**DOI:** 10.1007/s00222-018-0816-y

**Published:** 2018-09-08

**Authors:** David Helm, Gilbert Moss

**Affiliations:** 1 Department of Mathematics, 155 South 1400 East, JWB 233, Salt Lake City, UT 84112 USA; 2 Department of Mathematics, South Kensington Campus, Imperial College London, London, SW7 2AZ UK

**Keywords:** 11F33, 11F70, 22E50

## Abstract

We prove a descent criterion for certain families of smooth representations of ${\text {GL}}_n(F)$ (*F* a *p*-adic field) in terms of the $\gamma$-factors of pairs constructed in Moss (Int Math Res Not 2016(16):4903–4936, 2016). We then use this descent criterion, together with a theory of $\gamma$-factors for families of representations of the Weil group $W_F$ (Helm and Moss in Deligne–Langlands gamma factors in families, arXiv:1510.08743v3, 2015), to prove a series of conjectures, due to the first author, that give a complete description of the center of the category of smooth $W(k)[{\text {GL}}_n(F)]$-modules (the so-called “integral Bernstein center”) in terms of Galois theory and the local Langlands correspondence. An immediate consequence is the conjectural “local Langlands correspondence in families” of Emerton and Helm (Ann Sci Éc Norm Supér (4) 47(4):655–722, 2014).

## Introduction

The *center* of an abelian category ${\mathcal {A}}$ is the ring of natural transformations from the identity functor of ${\mathcal {A}}$ to itself. It is a commutative ring that acts naturally on every object of ${\mathcal {A}}$, compatibly with all morphisms of ${\mathcal {A}}$.

In [1], Bernstein and Deligne study the center of the category of smooth complex representations of a *p*-adic reductive group *G*. In particular, they show that such a category is an infinite direct product of full subcategories (called *blocks*). For each such block they give a concrete and explicit description of the center of each block, and an explicit description of the action of this center on each irreducible object of the block.

In the context of modular representation theory, however, much less is known. Paskunas [12] gives a complete description of the center of the category of finite length representations of ${\text {GL}}_2({\mathbb Q}_p)$ over *p*-adic integer rings, and uses this description to describe the image of the Colmez functor. Beyond this, however, the only results currently in the literature are in [6], which describes the center of the category of smooth $W(k)[{\text {GL}}_n(F)]$-modules, where *F* is a *p*-adic field and *k* is an algebraically closed field of characteristic $\ell$ different from *p*. We will refer to the center of this category as the *integral Bernstein center* in what follows.

Ideally, one would like to have an analogue of the Bernstein–Deligne result for the integral Bernstein center; that is, an explicit description of the algebra itself, together with its action on simple smooth $W(k)[{\text {GL}}_n(F)]$-modules. Results of the first author in [6], show that although the integral Bernstein center has several nice properties, and can be explicitly described in small examples, it quickly becomes too complicated (particularly when *n* is large compared to $\ell$) to admit a description along the lines of Bernstein–Deligne.

Rather than seeking for descriptions of the integral Bernstein center in terms of explicit algebras, it turns out to be better to try to understand the integral Bernstein center via Galois theory and the local Langlands correspondence. A first step in this direction was taken in [7], which gives a conjectural description of completions of the integral Bernstein center at maximal ideals in terms of universal framed deformation rings of Galois representations. (For the precise statements, see Conjectures 7.5 and 7.6 of [7].)

This conjecture, if established, would have considerable arithmetic implications. For one thing, the main result of [7] ([7], Theorem 7.9) shows that this conjecture implies the conjectural “local Langlands correspondence in families” of [5]. Moreover, this conjecture allows one to construct elements of the integral Bernstein center by considering natural invariants of Galois representations. For instance, given an element *w* of $W_F$, there would be a unique element of the integral Bernstein center that acts on every irreducible admissible representation $\Pi$ in characteristic zero by the scalar ${\text {tr}}\rho (w)$, where $\rho$ is the representation of $W_F$ attached to $\pi$ via local Langlands. (Such elements were first constructed by Chenevier over fields of characteristic zero ([4], Proposition 3.11), and have also been considered by Scholze, again in characteristic zero, in [13].) Ila Varma has noted that the existence of such elements in the integral Bernstein center is useful for formulating local-global compatibility statements, for instance for torsion classes in the cohomology of Shimura varieties.

In [8], the first author further refined this conjecture to a statement about finite type algebras. In particular, for each block $\nu$ of the category of smooth representations of ${\text {GL}}_n$, let $Z_{\nu }$ denote its center. The construction of section 9 of [8] gives a pair $(R_{\nu },\rho _{\nu })$, where $R_{\nu }$ is a finite type *W*(*k*)-algebra, and $\rho _{\nu }: W_F \rightarrow {\text {GL}}_n(R_{\nu })$ is an *n*-dimensional representation of $W_F$. The *k*-points of ${\text {Spec}}R_{\nu }$ are in bijection with isomorphism classes of (suitably rigidified) *n*-dimensional representations of $W_F$ over *k* that correspond, via mod $\ell$ local Langlands, to objects of the block $\nu$. Moreover the completion of $R_{\nu }$ at any one of these *k*-points is a universal deformation of the corresponding representation. Thus $R_{\nu }$ is a finite type object that interpolates the deformation rings appearing in the conjecture of [7].

The paper [8] then formulates two conjectures relating $Z_{\nu }$ and $R_{\nu }$. The first of these, which we will henceforth call the “weak conjecture” is that there is a map from $Z_{\nu }$ to $R_{\nu }$ satisfying a certain compatibility with the characteristic zero local Langlands correspondence. The second conjecture (the so-called “strong conjecture”) explicitly describes the image of this map. (We refer the reader to Sect. 7 for a more detailed discussion of this; in particular the precise statements are given as Conjectures 7.2 and 7.3, below.) The strong conjecture is strictly stronger than the weak conjecture, and implies the earlier conjectures of [7] (and thus the “local Langlands correspondence in families” of [5]).

The main objective of this paper is to establish the “strong conjecture” of [8], and therefore all of the other conjectures. To do so we rely heavily on the main result of [8], which shows that if the “weak conjecture” holds for all ${\text {GL}}_m(F)$ with $m \le n$, then the “strong conjecture” holds for ${\text {GL}}_n(F)$. Our approach will be to assume the strong conjecture for ${\text {GL}}_{n-1}(F)$, and show that it implies the “weak conjecture” for ${\text {GL}}_n(F)$ (Theorem 7.5, below.) Since both conjectures follow easily from local class field theory for ${\text {GL}}_1$, a straightforward induction then gives the desired result.

Our proof of Theorem 7.5 is modeled on Henniart’s approach to the *n* by $n-1$ converse theorem [9]. In particular we require notions such as Whittaker functions, zeta integrals, and $\gamma$-factors for representations over base rings that can be arbitrary *W*(*k*)-algebras. The correct context for such a theory is the setting of so-called “co-Whittaker” modules, developed by the first author in [7]. We recall the definition and basic theory of these modules in Sect. 2 below. Of particular importance to us will be the “universal co-Whittaker modules”; these live over direct factors $Z_{\nu }$ of the integral Bernstein center, and every co-Whittaker module arises, up to a certain natural equivalence, by base change from a universal one. Section 3 develops some basic “descent” criteria for a co-Whittaker module over an algebra *A*; the key point is that such a module arises by base change from a subalgebra $A'$ if it admits “sufficiently many” Whittaker functions that take values in $A'$. (Theorem 3.2 below.) Our ultimate goal is to apply this theorem to a certain subalgebra $Z'_{\nu }$ of $Z_{\nu }$; the point is that if we can find sufficiently many Whittaker functions of the universal co-Whittaker module over $Z_{\nu }$ with values in $Z'_{\nu }$, then the universality will tell us that the identity map on $Z_{\nu }$ factors through $Z'_{\nu }$, so that $Z'_{\nu }$ is in fact all of $Z_{\nu }$.

The subalgebra $Z'_{\nu }$ in question will be constructed using $\gamma$-factors of pairs. In [11], the second author developed a theory of zeta integrals and $\gamma$-factors for co-Whittaker modules that is compatible with the classical theory over algebraically closed fields of characteristic zero and that satisfies a suitable local functional equation. We recall this theory in Sect. 4 below.

In Sect. 5 we prove the key technical result of the paper, Theorem 5.1, which reformulates our descent theorem for co-Whittaker modules in terms of $\gamma$-factors of pairs. In particular, we suppose we have a co-Whittaker $A[{\text {GL}}_n(F)]$-module *V*, and a subalgebra $A'$ of *A* such that *A* is finitely generated as an $A'$ module. For each direct factor $Z_{\nu '}$ of the integral Bernstein center for ${\text {GL}}_{n-1}(F)$, we have a corresponding universal co-Whittaker module $W_{\nu '}$. We show that if the $\gamma$-factors attached to the pairs $V \times W_{\nu '}$ (a priori formal Laurent series with coefficients in $A \otimes Z_{\nu '}$) have coefficients in $A' \otimes Z_{\nu '}$, then *V* arises via base change from a co-Whittaker module over $A'$.

Applying this with $A = Z_{\nu }$ and *V* the universal co-Whittaker module over *A* lets us prove that, after completing at a maximal ideal, $Z_{\nu }$ is generated by a suitable set of elements derived from coefficients of “universal $\gamma$-factors of pairs”. Although we do not make direct use of this, we include this in Sect. 6 as a result of independent interest.

In Sect. 7 we prove our main result, by using Theorem 5.1 to show that the strong conjecture for ${\text {GL}}_{n-1}(F)$ implies the weak conjecture of ${\text {GL}}_n(F)$. Two additional ingredients are necessary. The first is that it is easy to show that the weak conjecture holds after inverting $\ell$, using the Bernstein–Deligne description of the Bernstein center for fields of characteristic zero (this is Theorem 10.4 of [8]). This gives us a map $Z_{\nu } \rightarrow R_{\nu }[\frac{1}{\ell }]$, compatible with local Langlands; the problem is then to show that its image lies in $R_{\nu }$. Similarly, the strong conjecture for ${\text {GL}}_{n-1}(F)$ gives us maps $Z_{\nu '} \rightarrow R_{\nu '}$ for each factor $Z_{\nu '}$ of the integral Bernstein center of ${\text {GL}}_{n-1}(F)$, whose image we have control over. Since these maps are compatible with local Langlands, they take the $\gamma$-factor of the pair $W_{\nu } \times W_{\nu '}$ to a Laurent series with coefficients in $R_{\nu }[\frac{1}{\ell }] \otimes R_{\nu '}$ that specializes, at any characteristic zero point *x*, to the $\gamma$-factor of the specialization of $\rho _{\nu } \otimes \rho _{\nu '}$ at *x*.

The second key ingredient is the work of [10], where we show that given a family $\rho$ of Galois representations over a suitable base *R*, there is a unique Laurent series with coefficients in *R* that interpolates the $\gamma$-factors of $\rho$ at characteristic zero points. This means that the image of the $\gamma$-factor of $W_{\nu } \times W_{\nu '}$ actually lies in $R_{\nu } \otimes R_{\nu '}$. In particular, if $Z'$ denotes the preimage of $R_{\nu }$ under the map $Z_{\nu '} \rightarrow R_{\nu }[\frac{1}{\ell }]$, then the coefficients of every universal $\gamma$-factor lie in $Z' \otimes R_{\nu '}$. Theorem 5.1 then shows that $Z' = Z_{\nu }$, completing the proof.

## Co-Whittaker modules

The appropriate context for the study of converse theorems in modular representation theory was developed by the first author in  [7]. We begin by summarizing the relevant results.

Let *F* be a *p*-adic field, and let $G_n$ denote the group ${\text {GL}}_n(F)$. Let *k* be an algebraically closed field of characteristic $\ell$ different from *p*, and let *A* be a *W*(*k*)-algebra. Since *k* contains the *p*-power roots of unity, we can fix an additive character $\psi : F \rightarrow W(k)^{\times }$. We will also regard $\psi$ as a character of the subgroup $U_n$ of $G_n$ consisting of unipotent upper triangular matrices, via the formula

$$\begin{aligned} \psi (u) = \psi (u_{12} + \cdots + u_{n-1,n}). \end{aligned}$$

Since *A* is a *W*(*k*)-algebra, we will often regard $\psi$ as an *A*-valued character.

In this context we have an “*n*th derivative functor” $V \mapsto V^{(n)}$ from the category ${\text {Rep}}_A(G_n)$ of smooth $A[G_n]$-modules to the category of *A*-modules, that takes a smooth $A[G_n]$-module *V* to the module of $\psi$-coinvariants in *V*. We refer the reader to [5], section 3.1, for basic properties of the functor $V \mapsto V^{(n)}$; in particular this functor is exact, and we have a natural isomorphism

$$\begin{aligned} (V \otimes _A M)^{(n)} \cong V^{(n)} \otimes _A M \end{aligned}$$

for any *A*-module *M*.

Central to our approach is the notion of a *co-Whittaker* $A[G_n]$-*module*, defined below:

### Definition 2.1

([7], 6.1) A smooth $A[G_n]$-module *V* is co-Whittaker if the following conditions hold:

1. *V* is admissible as an $A[G_n]$-module,
2. $V^{(n)}$ is a free *A*-module of rank one, and
3. if *W* is a quotient of *V* such that $W^{(n)} = 0$, then $W = 0$.

(This is not quite the definition given in [7], but is easily seen to be equivalent, for instance by using Lemma 3.4 of [7].)

Conditions (1)–(3) above imply easily that for any co-Whittaker $A[G_n]$-module *V*, the map $A \rightarrow {\text {End}}_{A[G_n]}(V)$ is an isomorphism (c.f. [7], Proposition 6.2).

If *V* and $V'$ are co-Whittaker $A[G_n]$-modules, we say that *V* *dominates* $V'$ if there is a surjection $V \rightarrow V'$ that induces an isomorphism of $V^{(n)}$ with $(V')^{(n)}$. This induces an equivalence relation on the set of isomorphism classes of co-Whittaker $A[G_n]$-modules, in which *V* and $V'$ are equivalent if there exists a co-Whittaker module $V''$ that dominates both *V* and $V'$.

In [7] there is constructed a “universal” co-Whittaker module up to this notion of equivalence. The key tool is the integral Bernstein center for ${\text {GL}}_n(F)$; that is, the center of the category ${\text {Rep}}_{W(k)}(G_n)$.

Recall that for an abelian category ${\mathcal {A}}$, the *center* of ${\mathcal {A}}$ is the ring of natural transformations from the identity functor on ${\mathcal {A}}$ to itself. This ring acts naturally on every object in ${\mathcal {A}}$. We denote by $Z_n$ the center of ${\text {Rep}}_{W(k)}(G_n)$.

A primitive idempotent *e* of $Z_n$ gives rise to a direct factor $e{\text {Rep}}_{W(k)}(G_n)$; this is the full subcategory of ${\text {Rep}}_{W(k)}(G_n)$ on which *e* acts as the identity. The primitive idempotents of $Z_n$ were described in [6]; they are in bijection with inertial equivalence class of pairs $(L,\pi )$, where *L* is a Levi subgroup of $G_n$ and $\pi$ is an irreducible supercuspidal *k*-representation of *L*. If *e* is the idempotent corresponding to the pair $(L,\pi )$, then a representation *V* in ${\text {Rep}}_{W(k)}(G_n)$ lies in $e{\text {Rep}}_{W(k)}(G_n)$ if, and only if, every simple subquotient of *V* has *mod* $\ell$ *inertial supercuspidal support* given by $(L,\pi )$ (in the sense of [6], Definition 4.12).

The center $Z_n$ decomposes as a product, over the primitive idempotents *e*, of the rings $eZ_n$. The structure of these rings was investigated extensively in [6]; in particular, we have:

### Theorem 2.2

([6], Theorem 10.8) The ring $eZ_n$ is a finitely generated, reduced, $\ell$-torsion free *W*(*k*)-algebra.

Let $W_n$ be the smooth $W(k)[G_n]$-module ${\text {c-Ind}}_{U_n}^{G_n} \psi$. Then for any primitive idempotent *e* of $Z_n$, we have an action of $eZ_n$ on $eW_n$. We then have:

### Theorem 2.3

([7], Theorem 6.3) The smooth $eZ_n[G_n]$-module $eW_n$ is a co-Whittaker $eZ_n[G_n]$-module.

Now let *A* be a *W*(*k*)-algebra, and let *V* be a co-Whittaker *A*[*G*]-module. Suppose further that *V* lies in $e{\text {Rep}}_{W(k)}(G_n)$ for some primitive idempotent *e*. Then $eZ_n$ acts on *V*, and since every endomorphism of *V* is a scalar ([7, Prop 6.2]), this action is given by a map $f_V: eZ_n \rightarrow A$. Note that if *V* dominates $V'$, then the maps $f_V$ and $f_{V'}$ coincide.

In the converse direction, we have:

### Theorem 2.4

([7], Theorem 6.3) If *A* is Noetherian and has an $eZ_n$-algebra structure, the module $eW_n \otimes _{eZ_n} A$ is a co-Whittaker $A[G_n]$-module that dominates *V*.

In particular the maps $f_V$ and $f_{V'}$ coincide if, and only if, *V* is equivalent to $V'$, and in this case both *V* and $V'$ are dominated by $eW_n \otimes _{eZ_n} A$. We thus say that, up to the equivalence relation induced by dominance, $eW_n$ is the universal co-Whittaker module contained in $e{\text {Rep}}_{W(k)}(G_n)$.

The smooth dual of a co-Whittaker module is rarely itself a co-Whittaker module. However, there is a natural “duality” operation on equivalence classes of co-Whittaker modules. Indeed, following Gelfand-Kazhdan, for *any* smooth $W(k)[G_n]$-module *V*, we denote by $V^{\iota }$ the $W(k)[G_n]$-module with the same underlying set as *V*, but for which the action of $g \in G_n$ on $V^{\iota }$ coincides with the action of $(g^t)^{-1}$ on *V*. The functor $V \mapsto V^{\iota }$ is left exact and covariant, and, for any *V*, the *W*(*k*)-modules $V^{(n)}$ and $(V^{\iota })^{(n)}$ are isomorphic. Moreover, $W_n^{\iota }$ is isomorphic to $W_n$. (The latter two claims are immediate from the fact that the character $\psi$ of $U_n$ is conjugate to the character $u \mapsto \psi ^{-1}(u^t)$ of the “opposite” unipotent group $\overline{U}_n$.) In particular, if *V* is a co-Whittaker $A[G_n]$-module, then so is $V^{\iota }$.

Moreover, Bernstein–Zelevinski show that if *V* is an irreducible smooth $\overline{{\mathcal K}}[G_n]$-module, then $V^{\iota }$ is simply the smooth dual of *V* (this follows immediately from [2, Thm 7.3]).

From this we deduce:

### Proposition 2.5

Let *e* be a primitive idempotent of $Z_n$. Then there exists a primitive idempotent $e^{\iota }$ of $Z_n$ such that an irreducible $\overline{{\mathcal K}}$ representation of $G_n$ lies in $e{\text {Rep}}_{W(k)}(G)$ if, and only if, its smooth $\overline{{\mathcal K}}$-dual lies in $e^{\iota }{\text {Rep}}_{W(k)}(G)$. Moreover, there is a unique isomorphism:

$$\begin{aligned} x \mapsto x^{\iota }: e Z_n \rightarrow e^{\iota } Z_n \end{aligned}$$

such that for all irreducible $\overline{{\mathcal K}}$-representations $\pi$ in $e{\text {Rep}}_{W(k)}(G)$, the action of $eZ_n$ on $\pi$ is given by the map:

$$\begin{aligned} x \mapsto f_{\pi ^{\vee }}(x^{\iota }): eZ_n \rightarrow \overline{{\mathcal K}}. \end{aligned}$$

(Here $\pi ^{\vee }$ is the smooth dual of $\pi$.)

### Proof

We have seen that the map $Z_n \rightarrow {\text {End}}_{W(k)[G_n]}(W_n)$ is an isomorphism. As $W_n$ is isomorphic to $W_n^{\iota }$, we obtain an involution $z \mapsto z^{\iota }$ on $Z_n$ by identifying $Z_n$ with ${\text {End}}_{W(k)[G_n]}(W_n)$ and considering the involution $f \mapsto f^{\iota }$ on this space of endomorphisms. All of the claims above are now immediate from the basic properties of the functor $V \mapsto V^{\iota }$. $\square$

### Proposition 2.6

Let *V* be a co-Whittaker *A*[*G*]-module. Let $f_V^{\iota }: e^{\iota }Z_n \rightarrow A$ be the map defined by $f_V^{\iota }(x) = f_V(x^{\iota })$. Then $V^{\iota }$ is isomorphic to the co-Whittaker module $e^{\iota }W_n \otimes _{e^{\iota }Z_n, f_V^{\iota }} A$.

### Proof

This is clear, as $z \in Z_n$ acts on *V* by $f_V(z)$, hence on $V^{\iota }$ by $f_V(z)^{\iota }$. It thus suffices to show that the latter is equal to $f_V(z^{\iota })$. This is clear, though, by applying the functor $V \mapsto V^{\iota }$ to diagrams of the form:

$$\begin{aligned} \begin{array}{ccc} W_n &{} \rightarrow &{} W_n \\ \downarrow &{} &{} \downarrow \\ V &{} \rightarrow &{} V\\ \end{array} \end{aligned}$$

where the left-hand and right-hand maps $W_n \rightarrow V$ agree, the top map is multiplication by *z*, and the bottom map is multiplication by $f_V(z)$. $\square$

We regard the functor $V \mapsto V^{\iota }$ on co-Whittaker models as an operation that interpolates the “smooth dual” operation across a co-Whittaker family.

## Whittaker functions and Schwartz functions

Co-Whittaker modules are useful to us because they provide a natural context for studying the variation of Whittaker functions in families. In this section we recall the details of this theory.

For any smooth *A*[*G*]-module *V*, and any *A*-module *M*, Frobenius reciprocity gives an isomorphism:

$$\begin{aligned} {\text {Hom}}_{A}(V^{(n)},M) \cong {\text {Hom}}_{A[G]}(V, {\text {Ind}}_{U_n}^{G_n} \psi _M), \end{aligned}$$

where $\psi _M$ is a copy of *M* on which $U_n$ acts via $\psi$. We call an element *f* of ${\text {Hom}}_{A}(V^{(n)},M)$ an *M-valued Whittaker functional* on *V*. For any element *v* of *V*, the *M-valued Whittaker function* $w_f(v)$ attached to *f* and *v* is the element of ${\text {Ind}}_{U_n}^{G_n} \psi _M$ given by ${\tilde{f}}(v)$, where ${\tilde{f}}$ is the element of ${\text {Hom}}_{A[G]}(V,{\text {Ind}}_{U_n}^{G_n} \psi _M)$ corresponding to *f*. We will often regard $w_f(v)$ as a smooth function on $G_n$; explicitly, one has $w_f(v)(g) = f(\overline{gv})$, where $\overline{gv}$ is the image of *gv* in $V^{(n)}$.

We will most often make use of this when V is a co-Whittaker module over *A*, and *f* is an isomorphism $V^{(n)} \cong A$. In this case we obtain a map ${\tilde{f}}: V \rightarrow {\text {Ind}}_{U_n}^{G_n} \psi _A$. We denote the image of this map by ${\mathcal W}(V,\psi )$ and call it the *A-valued Whittaker model* of *V* with respect to $\psi$.

For a fixed *V* it is possible to construct Whittaker functions with prescribed values on a large subgroup of $G_n$. More precisely, let $P_n$ be the so-called “mirabolic subgroup” of $G_n$; that is, the subgroup of $G_n$ consisting of matrices whose last row is the vector $(0,\ldots ,0,1)$.

There are natural isomorphisms of functors:

$$\begin{aligned}&V^{(n)} \cong {\text {Hom}}_{W(k)[P_n]}({\text {c-Ind}}_{U_n}^{P_n} \psi , V)\\&{\text {Hom}}_{W(k)}(V^{(n)},W) \cong {\text {Hom}}_{W(k)[P_n]}(V,{\text {Ind}}_{U_n}^{P_n} \psi _W) \end{aligned}$$

due to Bernstein–Zelevinsky. More precisely, this follows from the existence of isomorphisms:

$$\begin{aligned}&(\Phi ^+)^{n-1}\Psi ^+ W(k) \cong {\text {c-Ind}}_{U_n}^{P_n} \psi \\&(\hat{\Phi }^+)^{n-1}\Psi ^+ W(k) \cong {\text {Ind}}_{U_n}^{P_n} \psi \end{aligned}$$

(notation as in [3], section 3) and the formalism of the Bernstein–Zelevinsky functors (developed in [3], section 3 over the complex numbers and in [5], section 3.1 over *W*(*k*).)

The first of these isomorphisms give rise to a natural map:

$$\begin{aligned} {\text {c-Ind}}_{U_n}^{P_n} \psi \otimes V^{(n)} \rightarrow V. \end{aligned}$$

Note that we may identify ${\text {c-Ind}}_{U_n}^{P_n} \psi \otimes V^{(n)}$ with ${\text {c-Ind}}_{U_n}^{P_n} \psi _{V^{(n)}}$, allowing us to view the domain of this map as a space of compactly supported $V^{(n)}$-valued functions on $P_n$. The image of this map is often called the space of *Schwartz functions* of *V*.

On the other hand, the identity map $V^{(n)} \rightarrow V^{(n)}$ gives rise to a map $V \rightarrow {\text {Ind}}_{U_n}^{G_n} \psi _{V^{(n)}}$. Composing with restriction to $P_n$ gives us a series of maps:

3.1 $$\begin{aligned} {\text {c-Ind}}_{U_n}^{P_n} \psi _{V^{(n)}} \rightarrow V \rightarrow {\text {Ind}}_{U_n}^{G_n} \psi _{V^{(n)}} \rightarrow {\text {Ind}}_{U_n}^{P_n} \psi _{V^{(n)}} \end{aligned}$$

and we have:

### Lemma 3.1

The composition of the chain of maps 3.1 is the natural inclusion:

$$\begin{aligned} {\text {c-Ind}}_{U_n}^{P_n} \psi _{V^{(n)}} \rightarrow {\text {Ind}}_{U_n}^{P_n} \psi _{V^{(n)}}. \end{aligned}$$

### Proof

This is a fairly easy consequence of the Bernstein–Zelevinski formalism. The composition of the maps:

$$\begin{aligned} V \rightarrow {\text {Ind}}_{U_n}^{G_n} \psi _{V^{(n)}} \rightarrow {\text {Ind}}_{U_n}^{P_n} \psi _{V^{(n)}} \end{aligned}$$

is simply the map $V \rightarrow {\text {Ind}}_{U_n}^{P_n} \psi _{V^{(n)}}$ attached, via Frobenius reciprocity, to the identity map on $V^{(n)}$. Translating this into the language of [3] via the isomorphisms $V^{(n)} \cong \Psi ^-(\Phi ^-)^{(n-1)} V$ and ${\text {Ind}}_{U_n}^{P_n} \psi _W \cong (\hat{\Phi }^+)^{(n-1)} \Psi ^+ W$ we find that, under these isomorphisms, this map corresponds to the map:

$$\begin{aligned} V \rightarrow (\hat{\Phi }^+)^{(n-1)} \Psi ^+ \Psi ^- (\Phi ^-)^{(n-1)} V \end{aligned}$$

which comes from the identity on $\Psi ^- (\Phi ^-)^{(n-1)} V,$ via the adjunctions of the pairs $\Phi ^-,{\hat{\Phi }}^+$, and $\Psi ^-,\Psi ^+$.

Similarly, under the isomorphism of ${\text {c-Ind}}_{U_n}^{P_n} \psi _W$ with $(\Phi ^+)^{(n-1)}\Psi ^+ W$, the map ${\text {c-Ind}}_{U_n}^{P_n} \psi _{V^{(n)}} \rightarrow V$ is the map:

$$\begin{aligned} (\Phi ^+)^{(n-1)} \Psi ^+ \Psi ^- (\Phi ^-)^{(n-1)} V \rightarrow V \end{aligned}$$

corresponding to the identity on $\Psi ^- (\Phi ^-)^{(n-1)} V$ via the adjunction of $\Phi ^-$ and $\Phi ^+$.

It follows that the composition of these two maps is given by the inclusion of $(\Phi ^+)^{(n-1)}$ into $({\hat{\Phi }}^+)^{(n-1)}$, and, under the identifications we have made, this inclusion corresponds in turn to the inclusion of ${\text {c-Ind}}_{U_n}^{P_n} \psi _{V^{(n)}}$ in ${\text {Ind}}_{U_n}^{P_n} \psi _{V^{(n)}}$. $\square$

It is immediate from Lemma 3.1 that when *V* is a co-Whittaker *A*[*G*]-module, and we fix an isomorphism *f* of $V^{(n)}$ with *A*, then given any function *h* in ${\text {c-Ind}}_{U_n}^{P_n} \psi _A$, there exists an element *v* of *V* such that the restriction of $w_f(v)$ to $P_n$ is equal to *h*.

Our main interest in the Schwartz functions and Whittaker functions comes from the following “descent” result for co-Whittaker modules:

### Theorem 3.2

Let *V* be a co-Whittaker *A*[*G*]-module in $e{\text {Rep}}_{W(k)}(G)$, and fix an isomorphism $f: V^{(n)} \cong A$. Let $A'$ be a *W*(*k*)-subalgebra of *A*, and suppose that the composition:

$$\begin{aligned} {\text {c-Ind}}_{U_n}^{P_n} \psi _{A'} \rightarrow {\text {c-Ind}}_{U_n}^{P_n} \psi _A \rightarrow V \rightarrow {\text {Ind}}_{U_n}^{G_n} \psi _A \end{aligned}$$

has image contained in ${\text {Ind}}_{U_n}^{G_n} \psi _{A'}$ (i.e. the “$A'$-valued Schwartz functions of *V* have $A'$-valued Whittaker functions”). Then the map $eZ_n \rightarrow A$ giving the action of $eZ_n$ on *V* factors through $A'$.

### Proof

Let $V'$ be the preimage of ${\text {Ind}}_{U_n}^{G_n} \psi _{A'}$ under the map

$$\begin{aligned} V \rightarrow {\text {Ind}}_{U_n}^{G_n} \psi _A. \end{aligned}$$

The map $V' \rightarrow {\text {Ind}}_{U_n}^{G_n} \psi _{A'}$ gives rise to a map $(V')^{(n)} \rightarrow A'$ that fits into a commutative diagram:

$$\begin{aligned} \begin{array}{ccc} V'^{(n)} &{} \rightarrow &{} A'\\ \downarrow &{} &{} \downarrow \\ V^{(n)} &{} \rightarrow &{} A \end{array} \end{aligned}$$

in which the left-hand vertical map is injective (by exactness of the derivative) and the bottom horizontal map is the isomorphism *f*. We therefore have an injection of $(V')^{(n)}$ into $A'$.

On the other hand, our assumptions above show that the image of ${\text {c-Ind}}_{U_n}^{P_n} \psi _{A'}$ in *V* is contained in $V'$; the map

$$\begin{aligned} {\text {c-Ind}}_{U_n}^{P_n} \psi _{A'} \rightarrow V' \end{aligned}$$

gives rise to a map $A' \rightarrow (V')^{(n)}$ whose composition with the injection of $(V')^{(n)}$ into $A'$ is the identity. The isomorphism $V^{(n)} \rightarrow A$ thus identifies $(V')^{(n)}$ with $A'$.

The ring $eZ_n$ acts $A'$-linearly on $V'$ and hence on $(V')^{(n)}$; this action is given by a map $eZ_n \rightarrow A'$. Since $(V')^{(n)}$ generates $V^{(n)}$ as an *A*-module, and the map $(V')^{(n)} \rightarrow V^{(n)}$ is $eZ_n$-equivariant, the action of $eZ_n$ on $V^{(n)}$ is via this same map, and the result is now immediate. $\square$

In light of this result it will be useful to develop criteria for when a Whittaker function of *V* takes values in a given subalgebra $A'$ of *A*. We first note:

### Lemma 3.3

Let $A'$ be a *W*(*k*)-subalgebra of *A*, and let *h* be a function in the compact induction ${\text {c-Ind}}_{U_n}^{G_n} \psi _A$. Suppose that for all primitive idempotents *e* of $Z_n$, and all functions *g* in $e{\text {c-Ind}}_{U_n}^{G_n} \psi ^{-1}$, the integral

$$\begin{aligned} \int _{U_n{\setminus } G_n} h(x)g(x) d\mu (x) \end{aligned}$$

takes values in $A'$. Then *h* lies in ${\text {c-Ind}}_{U_n}^{G_n} \psi _{A'}$.

### Proof

As ${\text {c-Ind}}_{U_n}^{G_n} \psi ^{-1}$ is the direct sum, over all primitive *e*, of $e{\text {c-Ind}}_{U_n}^{G_n} \psi ^{-1}$, it suffices to show, for any *h* that does *not* take values in $A'$, there exists a $g \in {\text {c-Ind}}_{U_n}^{G_n} \psi ^{-1}$ such that the integral of *h*(*x*)*g*(*x*) does not lie in $A'$. Fix such an *h*, and a compact open subgroup *K* of $G_n$ such that *h* is fixed under right translation by all $k \in K$. Then (since the values of $\psi$ lie in $A'$) there is an element *x* of $G_n$ such that *h* takes values in $A {\setminus } A'$ on the double coset $U_n x K$. On the other hand, for *K* sufficiently small there exists $g: U_n x K \rightarrow A'$ such that $g(uxk) = \psi ^{-1}(u)$ for all $u \in U_n$, $k \in K$. It is then clear that the integral of *h*(*x*)*g*(*x*) is valued in $A {\setminus } A'$. $\square$

We will use the fact that the spaces $e{\text {c-Ind}}_{U_n}^{G_n} \psi ^{-1}$ are co-Whittaker modules to convert this into a criterion that involves integration against Whittaker functions. Let *A* and *B* be *W*(*k*)-algebras, let *h* be an element of ${\text {c-Ind}}_{U_n}^{G_n} \psi _A$, and let *V* be a co-Whittaker $B[G_n]$-module. Fixing an isomorphism $f: V^{(n)} \cong B$ and an element *v* of *V* gives us a Whittaker function $w_f(v)$ in ${\text {Ind}}_{U_n}^{G_n} \psi _B$. Given any Whittaker function *w* in ${\mathcal W}(V,\psi )$ we can define the dual Whittaker function ${\tilde{w}}$ by ${\tilde{w}}(g) = w(\delta _n(g^t)^{-1})$, where $\delta _n$ is the matrix in $G_n$ whose only nonzero entries are 1’s along the antidiagonal. We have:

### Proposition 3.4

Let *w* lie in ${\mathcal W}(V,\psi )$. Then $\tilde{w}$ lies in ${\mathcal W}(V^{\iota },\psi ^{-1})$.

### Proof

This is clear, as $G_n$ acts on Whittaker functions by right translation, so one has $\widetilde{gw} = (g^t)^{-1}\tilde{w}$ for all *g*. $\square$

Let $\tilde{w}_f(v)$ be the dual Whittaker function to $w_f(v)$. Then the integral

$$\begin{aligned} \int _{U_n{\setminus } G_n} h(x) \otimes \tilde{w}_f(v)(x) d\mu (x) \end{aligned}$$

makes sense as an element of $A \otimes B$.

This is particularly useful in the case where $B = eZ_n$ and *V* is the universal co-Whittaker module $eW_n$. In this case one has some useful additional structure. First, regarding elements of $W_n = {\text {c-Ind}}_{U_n}^{G_n} \psi$, we see that “evaluation at the identity” gives a canonical map $V^{(n)} \rightarrow W(k)$ that corresponds to the inclusion of ${\text {c-Ind}}_{U_n}^{G_n} \psi$ in ${\text {Ind}}_{U_n}^{G_n} \psi$. We denote this map by $\theta _{e,n}$.

We also note that there is a natural isomorphism: ${\text {End}}_{W(k)[G]}(eW_n) \cong eW_n^{(n)}$. Since $eW_n$ is a co-Whittaker $eZ_n[G]$-module, its endomorphisms are canonically isomorphic to $eZ_n$, and we thus canonically identify

$$\begin{aligned} eZ_n \cong {\text {End}}_{W(k)[G]}(eW_n) \cong eW_n^{(n)}. \end{aligned}$$

In particular we can (and do) regard $\theta _{e,n}$ as a *W*(*k*)-linear map $eZ_n \rightarrow W(k)$.

Let *f* be the above isomorphism $(eW_n)^{(n)}\cong eZ_n$. Immediately from the definitions one finds that for $v \in eW_n$ and $x \in G$, one has $v(x) = \theta _{e,n}(w_f(v)(x))$; that is, one can recover the function $v: G_n \rightarrow W(k)$ from the $eZ_n$-valued Whittaker function of *v*. Similarly, one has $\tilde{v}(x) = \theta _{e,n}(\tilde{w}_f(v)(x))$, where $\tilde{v}(x)=v(\delta _n(g^t)^{-1})$.

From this, together with Lemma 3.3, we immediately deduce:

### Proposition 3.5

Let $A'$ be a *W*(*k*)-subalgebra of *A*, and let *h* be a function in ${\text {c-Ind}}_{U_n}^{G_n} \psi _A$. Suppose that for all primitive idempotents *e* of $Z_n$, and all functions *g* in $e{\text {c-Ind}}_{U_n}^{G_n} \psi$, the integral

$$\begin{aligned} I(h,g) = \int _{U_n{\setminus } G_n} h(x) \otimes \tilde{w}_f(g) (x) d\mu (x) \end{aligned}$$

(an element of $A \otimes eZ_n$) satisfies $(1_A \otimes \theta _{e,n})(I(h,g)) \in A'$. Then *h* lies in ${\text {c-Ind}}_{U_n}^{G_n} \psi _{A'}$.

Another useful observation, also immediate from the above, is:

### Corollary 3.6

Let $A'$ be a *W*(*k*)-subalgebra of *A*, and let *h* be a function in ${\text {c-Ind}}_{U_n}^{G_n} \psi _A$. Suppose that for all primitive idempotents *e* of $Z_n$, and all functions *g* in $e{\text {c-Ind}}_{U_n}^{G_n} \psi$, the integral *I*(*h*, *g*) defined above lies in $A' \otimes eZ_n$. Then *h* lies in ${\text {c-Ind}}_{U_n}^{G_n} \psi _{A'}$.

## Zeta integrals and $\gamma$-factors

We now recall recent work of the second author [11] that constructs zeta integrals and $\gamma$-factors attached to pairs of co-Whittaker modules. Let $A_1$ and $A_2$ be Noetherian *W*(*k*)-algebras, and let $V_1$, $V_2$ be a co-Whittaker $A_1[G_n]$-module, and a co-Whittaker $A_2[G_{n-1}]$-module, respectively. Let *R* denote the *W*(*k*)-algebra $A_1 \otimes A_2$. Fixing isomorphisms of $V_1^{(n)}$ with $A_1$ and $V_2^{(n)}$ with $A_2$ gives us Whittaker functionals on $V_1$ and $V_2$.

For elements $w_1$ of ${\mathcal W}(V_1,\psi )$ and $w_2$ of ${\mathcal W}(V_2,\psi ^{-1})$ we define the “zeta integral” $\Psi (w_1,w_2,X)$ to be the formal series $\sum _{m = -\infty }^{\infty } c_m X^m,$ where the coefficient $c_m$ is given by the integral:

$$\begin{aligned} c_m := \int _{U_{n-1} {\setminus } \{g \in G_{n-1}: v(\det g) = m\}} \left( w_1({\begin{matrix} g &{} 0\\ 0 &{} 1\end{matrix}}) \otimes w_2(g)\right) dg. \end{aligned}$$

By [11], Lemma 2.1, $\Psi (w_1,w_2,X)$ is a well-defined element of $R[[X]] [X^{-1}]$. Note that the formation of zeta integrals is compatible with base change, in the following sense: if we have a map $f:A_1 \rightarrow B_1$, then the function $f(w_1)$ defined by $f(w_1)(x) = f(w_1(x))$ is an element of ${\mathcal W}(V \otimes _{A_1,f} B_1,\psi )$, and if $\Psi (w_1,w_2,X)$ is given by $\sum c_i X^i$, then $\Psi (f(w_1),w_2,X)$ is given by $\sum (f \otimes 1)(c_i) X^i$. A similar statement holds for base change via maps $A_2 \rightarrow B_2$.

A key point of [11] is a rationality result for such zeta integrals. Let *S* be the multiplicative system in $R[X,X^{-1}]$ consisting of all polynomials whose leading and trailing coefficients are units. Such polynomials are units in $R[[X]][X^{-1}]$, and nonzerodivisors in $R[X,X^{-1}]$. We therefore obtain an embedding:

$$\begin{aligned} S^{-1}R[X,X^{-1}] \rightarrow R[[X]][X^{-1}]. \end{aligned}$$

When *R* is a Noetherian ring, the following lemma characterizes the image of $S^{-1}R[X,X^{-1}]$ in $R[[X]][X^{-1}]$:

### Lemma 4.1

Suppose *R* is Noetherian. Let *f* be an element of $R[[X]][X^{-1}]$, and let $M_f$ be the $R[X,X^{-1}]$-submodule of $R[[X]][X^{-1}]/R[X,X^{-1}]$ generated by *f*. Then *f* lies in $S^{-1}R[X,X^{-1}]$ if, and only if, $M_f$ is a finitely generated *R*-module.

### Proof

Suppose that $M_f$ is a finitely generated *R*-module. Then there exists a positive integer *d* such that both $X^{-1}f$ and $X^d f$ are in the *R*-submodule of $M_f$ generated by $f,Xf, \ldots , X^{d-1}f$. We may thus write $X^{-1}f = P(X) f$ and $X^d f = Q(X)f$ where *P* and *Q* lie in *R*[*X*] and have degree at most $d-1$. Then $X^{-1} - P(X) - Q(X) + X^d$ annihilates *f* in $M_f$ and lies in *S*. Thus *f* lies in $S^{-1}R[X,X^{-1}]$.

Conversely, suppose *f* lies in $S^{-1}R[X,X^{-1}]$. Then there is an element of *S* that annihilates *f* in $M_f$, which we may take, without loss of generality, to be a polynomial of degree *d*. Then $M_f$ is spanned over *R* by $f, Xf, \ldots , X^{d-1}f$.$\square$

### Corollary 4.2

Let $R'$ be a Noetherian *W*(*k*)-subalgebra of *R* such that *R* is finitely generated as an $R'$-module. Let $S'$ be the subset of $R'[X,X^{-1}]$ consisting of polynomials whose first and last nonzero coefficients are units in $R'$. Then $(S')^{-1}R'[X,X^{-1}]$ is the intersection, in $R[[X]][X^{-1}]$, of the subrings $R'[[X]][X^{-1}]$ and $S^{-1}R[X,X^{-1}]$.

### Proof

It is clear that $(S')^{-1}R'[X,X^{-1}]$ is contained in this intersection. Conversely, suppose *f* is in this intersection. Then $M_f$ is finitely generated over *R*, and hence finitely generated over $R'$. By Lemma 4.1, if $M'_f$ is the $R'[X,X^{-1}]$-submodule of $R'[[X]][X^{-1}]/R'[X,X^{-1}]$ generated by *f*, it suffices to show $M'_f$ is finitely generated over $R'$. But the natural map $M'_f\rightarrow M_f$ is injective: let *gf* be an element of the kernel, where *g* is a power series with coefficients in $R'$; then *gf* lies in $R[X,X^{-1}]$ and also in $R'[[X]][X^{-1}]$ and thus must be in $R'[X,X^{-1}]$, so was already zero in $M'_f$. Since $R'$ is Noetherian, the finiteness of $M_f$ implies the finiteness of $M_f'$. $\square$

The rationality result of [11] now states:

### Theorem 4.3

([11], Theorem 2.2) For any $w_1 \in {\mathcal W}(V_1,\psi )$ and $w_2 \in {\mathcal W}(V_2,\psi ^{-1})$, the zeta integral $\Psi (w_1,w_2,X)$ lies in $S^{-1}R[X,X^{-1}]$.

The zeta integrals attached to pairs of co-Whittaker modules satisfy a functional equation generalizing the functional equation over the complex numbers:

### Theorem 4.4

([11], Theorem 3.4) There exists a unique element $\gamma (V_1 \times V_2, X, \psi )$ of $S^{-1}R[X,X^{-1}]$ such that, for all $w_1$ in ${\mathcal W}(V_1,\psi )$ and $w_2$ in ${\mathcal W}(V_2,\psi ^{-1})$, one has:

$$\begin{aligned} \Psi (w_1,w_2,X)\gamma (V_1 \times V_2, X, \psi ) \omega _{V_2}(-1)^{(n-1)} = \Psi ({\tilde{w}_1}, {\tilde{w}_2}, X^{-1}). \end{aligned}$$

(Here $\omega _{V_2}$ is the central character of $V_2$, viewed as taking values in $A_2$.)

Note that the compatibility of zeta integrals with base change, together with uniqueness of $\gamma$-factors, immediately implies a similar compatbility of $\gamma$-factors with extension of scalars.

Since the restriction $$w_1\left( {\begin{matrix}g&{}0\\ 0&{}1 \end{matrix}}\right) $$ is not in general compactly supported mod $U_{n-1}$, we must restrict attention to the individual coefficients $c_m$ before applying the descent result of Proposition 3.5. The following Lemma allows this to work.

### Lemma 4.5

If *V* is a co-Whittaker $A[G_n]$-module and $w\in \mathcal {W}(V,\psi )$, then for each integer *r* there exists a compact subset *C* of $G_{n-1}$ such that if $g\in G_{n-1}$ satisfies $v_F(\det (g))=r$ and $$w\left( {\begin{matrix}g&{}0\\ 0&{}1 \end{matrix}}\right) \ne 0$$, then $g\in U_{n-1}C$.

### Proof

This is the analog for co-Whittaker families of [9] [Lemme 2.4.2]. While a direct proof probably exists, we may deduce it formally using extension of scalars from $Z_n$. Without loss of generality suppose $V=eV$ for a primitive idempotent *e* of $Z_n$ and let $f_V:eZ_n\rightarrow A$ be the action of the Bernstein center. Choose $w'\in \mathcal {W}(eW_n,\psi )$ an $eZ_n$-valued Whittaker function such that $f_V\circ w' = w$ (cf. Theorem 2.4). If $g\in G_{n-1}$ is such that $$w\left( {\begin{matrix}g&{}0\\ 0&{}1 \end{matrix}}\right) \ne 0$$, then $$w'\left( {\begin{matrix}g&{}0\\ 0&{}1 \end{matrix}}\right) \ne 0$$, so it suffices to prove the lemma when $V=eW_n$ and $A=eZ_n$. Fix an integer *r*. For each minimal prime ${\mathfrak p}$ of $eZ_n$, the residue field $\kappa ({\mathfrak p})$ has characteristic zero and is a finite extension of the fraction field of *W*(*k*), so we may choose an isomorphism $\overline{\kappa ({\mathfrak p})}\cong {\mathbb C}$ and apply [9][Lemme 2.4.2] to obtain a compact subset $C_{{\mathfrak p}}$ satisfying the conclusion of the Lemma for $V\otimes _{eZ_n}\overline{\kappa ({\mathfrak p})}$. Since $eZ_n$ is reduced, if $$w'\left( {\begin{matrix}g&{}0\\ 0&{}1 \end{matrix}}\right) $$ is nonzero it remains nonzero in some $\kappa ({\mathfrak p})$, and so $g\in C_{{\mathfrak p}}$ for some ${\mathfrak p}$. We can therefore take $C=\bigcup _{{\mathfrak p}}C_{{\mathfrak p}}$. $\square$

## A descent result

Now fix a Noetherian *W*(*k*)-algebra *A*, and a co-Whittaker $A[G_n]$-module *V* in $e{\text {Rep}}_{W(k)}(G_n)$. For each idempotent $e'$ of $Z_{n-1}$, the module $e'W_{n-1}$ is a co-Whitaker $e'Z_{n-1}[G_{n-1}]$-module, and one can form the rational function $\gamma (V \times e'W_{n-1}, X^{-1}, \psi )$. If we expand this as a power series in *X*, then the coefficients of this power series lie in $A \otimes e'Z_{n-1}$.

Fix a *W*(*k*)-subalgebra $A'$ of *A*. Compatibility of $\gamma$-factors with base change implies that if *V* arises by base change from $A'$, then the coefficients of $\gamma (V \times e'W_{n-1}, X^{-1}, \psi )$ and $\gamma (V^{\iota }\times e'W_{n-1},X,\psi ^{-1})$ lie in $A' \otimes e'Z_{n-1}$. The objective of this section is to prove a partial converse to this theorem.

### Theorem 5.1

Suppose that *A* is finitely generated as an $A'$-module, and let *V* be a co-Whittaker $A[G_n]$-module such that for all $e'$, the coefficients of $\gamma (V \times e'W_{n-1}, X^{-1}, \psi )$ and $\gamma (V^{\iota }\times e'W_{n-1},X,\psi ^{-1})$ lie in $A' \otimes e'Z_{n-1}$. Then the map $f_V: eZ_n \rightarrow A$ giving the action of $eZ_n$ on *V* factors through $A'$.

Let *R* denote the ring $(A \otimes e'Z_{n-1})$, and let $R'$ denote the subalgebra $(A' \otimes e'Z_{n-1})$. Note that for all $e'$, $e'Z_{n-1}$ is flat over *W*(*k*), so that $R'$ is a subalgebra of *R*. As usual *S* and $S'$ will denote the multiplicative subsets of $R[X,X^{-1}]$ and $R'[X,X^{-1}]$ consisting of polynomials whose first and last coefficients are units. Toward proving Theorem 5.1 we first have:

### Proposition 5.2

With the hypotheses of Theorem 5.1, let *w* be an element of ${\mathcal W}(V,\psi )$. Then the restriction of *w* to $P_n$ takes values in $A'$ if and only if the restriction of ${\tilde{w}}$ to $P_n$ takes values in $A'$.

### Proof

Fix a primitive idempotent $e'$, and an element $w_2$ of ${\mathcal W}(e'W_{n-1},\psi ^{-1})$. The functional equation for $\Psi (w,{\tilde{w}_2}, X)$ reads:

$$\begin{aligned} \Psi (w,{\tilde{w}_2},X) \gamma (V \times (e')^{\iota }W_{n-1},X,\psi ) = \pm \Psi ({\tilde{w}}, w_2, X^{-1}). \end{aligned}$$

Applying the involution $X \mapsto X^{-1}$ of $S^{-1}R[X,X^{-1}]$ we obtain the identity:

$$\begin{aligned} \Psi (w,{\tilde{w}_2},X^{-1}) \gamma (V \times (e')^{\iota }W_{n-1},X^{-1},\psi ) = \pm \Psi ({\tilde{w}}, w_2, X). \end{aligned}$$

Since the zeta integral $\Psi (w,{\tilde{w}_2},X)$ depends, by definition, only on the restriction of *w* to $P_n$, this integral lies in $R'[[X]][X^{-1}]$. Since it also lies in $S^{-1}R[X,X^{-1}]$, and *R* is finitely generated over $R'$, we find that $\Psi (w,{\tilde{w}_2},X)$ lies in the subring $(S')^{-1}R'[X,X^{-1}]$ by Corollary 4.2, hence so does $\Psi (w,{\tilde{w}_2},X^{-1})$. The latter, considered as a power series in *X*, thus lies in $R'[[X]][X^{-1}]$. Therefore, so does $\Psi ({\tilde{w}}, w_2, X)$.

For each integer *r* denote by $h_r$ the function on $G_{n-1}$ given by

$$\begin{aligned} h_r(g)&= 0\quad \text { if } v_F(\det (g))\ne r\\ h_r(g)&= \tilde{w}\left( {\begin{matrix}g&{}0\\ 0&{}1 \end{matrix}}\right) \quad \text { if } v_f(\det (g))=r. \end{aligned}$$

The *r*’th coefficient of $\Psi ({\tilde{w}}, w_2, X,\psi )$ equals

$$\begin{aligned}\int _{U_{n-1}{\setminus } G_{n-1}} h_r(x) \otimes w_2(x) d\mu (x),\end{aligned}$$

and so this integral lies in $R'$. Therefore

$$\begin{aligned}(1 \otimes \theta _{e',n-1})\left( \int _{U_{n-1}{\setminus } G_{n-1}}h_r(x) \otimes w_2(x) d\mu (x)\right) \end{aligned}$$

lies in $A'$. By Lemma 4.5, $h_r$ is compactly supported modulo $U_{n-1}$. Since this is true for all $e'$ and all $w_2$, we conclude from Proposition 3.5 that $h_r$ takes values in $A'$. Since this is true for all *r* we conclude that $\tilde{w}$ takes values in $A'$ when restricted to $G_{n-1}$, and hence also to $P_n$.

The converse argument is nearly identical, starting with the functional equation

$$\begin{aligned} \Psi ({\tilde{w}},{\tilde{w}_2}, X) \gamma (V^{\iota } \times (e')^{\iota } W_{n-1},X,\psi ^{-1}) = \pm \Psi (w,w_2,X^{-1}). \end{aligned}$$

$$\square $$

Our main results are immediate consequences of this proposition:

### Corollary 5.3

With the hypotheses of Theorem 5.1, if *w* is an element of ${\mathcal W}(V,\psi )$ whose restriction to $P_n$ takes values in $A'$, then *w* takes values in $A'$ on all of $G_n$.

### Proof

The set of *w* whose restriction to $P_n$ takes values in $A'$ is clearly stable under $P_n$, and similarly the set of *w* such that the restriction of $\tilde{w}$ to $P_n$ takes values in $A'$ is stable under the transposed group $P_n^t$. Since $P_n$ and $P_n^t$ together generate all of $G_n$, the set of *w* whose restriction to $P_n$ takes values in $A'$ is stable under $G_n$. Hence any such *w* takes values in $A'$ on all of $G_n$.$\square$

Theorem 5.1 is now immediate from the previous corollary and Theorem 3.2.

## Generators for $eZ_n$

Although it will not be necessary for our main results, it is an interesting question, given a co-Whittaker $A[G_n]$-module *V*, to determine the image of the map $eZ_n \rightarrow A$ giving the action of the Bernstein center on *V* in terms of the $\gamma$-factors of *V*. One can try to approach this question via the techniques of the previous section, but the finiteness hypotheses that appear in places (stemming from Corollary 4.2) prevent us from being completely successful. We outline the basic ideas in this section. The approach is to construct elements of *A* that must lie in the image of the map $eZ_n \rightarrow A$.

If $\{c_i\}_{i\in {\mathbb Z}}$ are the coefficients of the power series $\gamma (V\times e'W_{n-1},X,\psi )$, and $\{d_i\}_{i\in {\mathbb Z}}$ are the coefficients of the power series $\gamma (V^{\iota } \times e'W_{n-1}, X^{-1}, \psi ^{-1})$, we define $\mathcal {S}$ to be the subset of *A* consisting of $(1 \otimes \theta _{e',n-1})(zc_i)$ and $(1 \otimes \theta _{e',n-1})(zd_i)$ for all $e'$, all $z\in e'Z_{n-1}$, and all $i\in {\mathbb Z}$.

If *V* arises by base change from a subring $A'\subset A$, then $A'$ must contain $\mathcal {S}$. Therefore, we proceed by fixing a *W*(*k*)-subalgebra $A'$ of *A* that contains the set $\mathcal {S}$.

### Lemma 6.1

Fix a primitive idempotent $e'$ of $Z_{n-1}$, and let $R = A \otimes e'Z_{n-1}$, $R' = A' \otimes e'Z_{n-1}$. Let $\Psi$ be any element of $R'[[X]][X^{-1}]$. Then for any coefficient *c* of $\Psi \cdot \gamma (V \times e'W_{n-1}, X^{-1}, \psi )$ or of $\Psi \cdot \gamma (V^{\iota } \times e'W_{n-1}, X^{-1}, \psi )$, the element $(1 \otimes \theta _{e',n-1})(c)$ of *A* lies in $A'$.

### Proof

The coefficient *c* is a sum of products of a cofficient of $\Psi$ and a coefficient of $\gamma (V \times e'W_{n-1},X,\psi )$, and therefore an $R'$-linear combination of coefficients of $\gamma$. Thus *c* is an $A'$-linear combination of elements of *R* of the form $zc_i$, where $z \in e'Z_{n-1}$ and $c_i$ is a coefficient of $\gamma (V \times e'W_{n-1},X,\psi )$. It follows (by *A*-linearity of $1 \otimes \theta _{e',n-1}$) that $(1 \otimes \theta _{e',n-1})(c)$ is an element of $A'$. $\square$

Then as a slight variation on Proposition 5.2 above, we obtain:

### Proposition 6.2

Suppose $A'$ is a *W*(*k*)-subalgebra of *A* containing $\mathcal {S}$, such that *A* is a finitely generated $A'$-module, and let *w* be an element of ${\mathcal W}(V,\psi )$. Then the restriction of *w* to $P_n$ takes values in $A'$ if, and only if, the restriction of ${\tilde{w}}$ to $P_n$ takes values in $A'$.

### Proof

Fix a primitive idempotent $e'$, and an element $w_2$ of $W(e'W_{n-1},\psi ^{-1})$. The functional equation for $\Psi (w,{\tilde{w}_2},X)$ reads:

$$\begin{aligned} \Psi (w,{\tilde{w}_2},X) \gamma (V \times (e')^{\iota }W_{n-1},X,\psi ) = \pm \Psi ({\tilde{w}}, w_2, X^{-1}). \end{aligned}$$

Applying the involution $X \mapsto X^{-1}$ of $S^{-1}R[X,X^{-1}]$ we obtain the identity:

$$\begin{aligned} \Psi (w,{\tilde{w}_2},X^{-1}) \gamma (V \times (e')^{\iota }W_{n-1},X^{-1},\psi ) = \pm \Psi ({\tilde{w}}, w_2, X). \end{aligned}$$

Since the zeta integral $\Psi (w,{\tilde{w}_2},X)$ depends, by definition, only on the restriction of *w* to $P_n$, this integral lies in $R'[[X]][X^{-1}]$. Since it also lies in $S^{-1}R[X,X^{-1}]$, and *R* is finitely generated over $R'$, we find that $\Psi (w,{\tilde{w}_2},X)$ lies in the subring $(S')^{-1}R'[X,X^{-1}]$, and hence so does $\Psi (w,{\tilde{w}_2},X^{-1})$. The latter, considered as a power series in *X*, thus lies in $R'[[X]][X^{-1}]$. Hence, by Lemma 6.1 above, if *c* is any coefficient of $\Psi ({\tilde{w}}, w_2, X)$, then $(1 \otimes \theta _{e',n-1})(c)$ lies in $A'$.

For each integer *r* denote by $h_r$ the function on $G_{n-1}$ given by

$$\begin{aligned} h_r(g)&= 0\quad \text { if } v_F(\det (g))\ne r\\ h_r(g)&= \tilde{w}\left( {\begin{matrix}g&{}0\\ 0&{}1 \end{matrix}}\right) \quad \text { if } v_f(\det (g))=r. \end{aligned}$$

Returning to the definition of the zeta integral, the *r*’th coefficient of $\Psi ({\tilde{w}}, w_2, X,\psi )$ equals

$$\begin{aligned} \int _{U_{n-1}{\setminus } G_{n-1}} h_r(x) \otimes w_2(x) d\mu (x), \end{aligned}$$

so we have shown that the integral

$$\begin{aligned} (1 \otimes \theta _{e',n-1})\left( \int _{U_{n-1}{\setminus } G_{n-1}}h_r(x) \otimes w_2(x) d\mu (x)\right) \end{aligned}$$

lies in $A'$. By Lemma 4.5, $h_r$ is compactly supported modulo $U_{n-1}$. Since this is true for all $e'$ and all $w_2$, we conclude from Proposition 3.5 that $h_r$ takes values in $A'$. Since this is true for all *r* we conclude that $\tilde{w}$ takes values in $A'$ when restricted to $G_{n-1}$, and hence also to $P_n$.

As before, the converse argument is nearly identical, starting with the functional equation

$$\begin{aligned} \Psi ({\tilde{w}},{\tilde{w}_2}, X) \gamma (V^{\iota } \times (e')^{\iota } W_{n-1},X,\psi ) = \pm \Psi (w,w_2,X^{-1}). \end{aligned}$$

$$\square $$

Just as in the previous section, we now find:

### Corollary 6.3

If $A'$ is a *W*(*k*)-subalgebra of *A* containing $\mathcal {S}$, such that *A* is a finitely generated $A'$-module, and *w* is an element of ${\mathcal W}(V,\psi )$ whose restriction to $P_n$ takes values in $A'$, then *w* takes values in $A'$ on all of $G_n$.

### Corollary 6.4

Suppose that $A'$ is a *W*(*k*)-subalgebra of *A* containing $\mathcal {S}$, and suppose that either

1. *A* is a finitely generated $A'$-module, or
2. *A* is a complete Noetherian local ring with residue field *k*, and $A'$ is closed in *A*.

Then the map $f_V: eZ_n \rightarrow A$ giving the action of $eZ_n$ on *V* factors through $A'$.

### Proof

When *A* is a finitely generated $A'$-module, this is immediate from the previous corollary and Theorem 3.2. When *A* is complete local with maximal ideal ${\mathfrak m}$, set $A_r = A/{\mathfrak m}^r$, let $A'_r$ be the image of $A'$ in $A_r$, and set $V_r = V \otimes _A A_r$. Then $A_r$ has finite length over *W*(*k*), and in particular is a finitely generated $A'_r$-module. Thus, for all *r*, the map $f_{V_r}$ factors through $A'_r$. Passing to the limit (and using the fact that $A'$ is closed in *A*), we find that $f_V$ factors through $A'$. $\square$

Now let ${\mathfrak m}$ be a maximal ideal of $eZ_n$ with residue field *k*. We then have:

### Corollary 6.5

The completion of $eZ_n$ at ${\mathfrak m}$ is toplogically generated by elements of the form $(1 \otimes \theta _{e',n-1})(zc_i)$ and $(1 \otimes \theta _{e',n-1})(zd_i)$, where $e'$ runs over the primitive idempotents of $Z_{n-1}$, *z* runs over the elements of $e'Z_{n-1}$, and $c_i$ and $d_i$ are the coefficients of the power series $\gamma (eW_n \times e'W_{n-1},X^{-1})$ and $\gamma (e^{\iota }W_n \times e'W_{n-1},X^{-1})$, respectively.

### Proof

Apply the previous corollary with *A* equal to the completion of $eZ_n$ at ${\mathfrak m}$, $V = eW_n \otimes _{eZ_n} A$, and $A'$ the closed subalgebra of *A* generated by the elements listed above. Then the map $eZ_n \rightarrow A$ factors through $A'$; since $eZ_n$ is dense in *A* the result follows. $\square$

## The local Langlands correspondence in families

We now turn to other applications of the descent technique. In particular, we recall Conjecture 1.3.1 of [5] (or rather, a slight reformulation of it in the spirit of section 7 of [7]):

### Conjecture 7.1

Let *A* be a reduced complete Noetherian local *W*(*k*)-algebra, flat over *W*(*k*), with residue field *k*, and let $\rho : G_F \rightarrow {\text {GL}}_n(A)$ be a continuous *n*-dimensional representation of the Galois group $G_F$. Then there exists a (necessarily unique) admissible $W(k)[G_n]$-module $\pi (\rho )$ such that:

1. $\pi (\rho )$ is *A*-torsion free,
2. $\pi (\rho )$ is a co-Whittaker *A*[*G*]-module, and
3. for each minimal prime ${\mathfrak a}$ of *A*, the representation $\pi (\rho )_{\mathfrak a}$ is $\kappa ({\mathfrak a})$-dual to the representation that corresponds to $\rho _{\mathfrak a}^{\vee }$ under the Breuil–Schneider generic local Langlands correspondence.

(Here $\kappa ({\mathfrak a})$ denotes the field of fractions of $A/{\mathfrak a}$, and $\rho _{\mathfrak a}^{\vee }$ is the $\kappa ({\mathfrak a})$-dual of $\rho _{\mathfrak a}$.)

The main result of [7] (Theorem 7.9) gives a reformulation of this conjecture in terms of the Bernstein center. More precisely, fix $\rho$ as in the conjecture and let ${\overline{\rho }}: G_F \rightarrow {\text {GL}}_n(k)$ be its residual representation. Let $R_{{\overline{\rho }}}^{\Box }$ denote the universal framed deformation ring of ${\overline{\rho }}$; in particular there is a map $\rho ^{\Box }: G_F \rightarrow {\text {GL}}_n(R_{{\overline{\rho }}}^{\Box })$ lifting ${\overline{\rho }}$ that is universal for lifts of ${\overline{\rho }}$ to complete Noetherian local *W*(*k*)-algebras with residue field *k*.

Then Conjecture 7.5 of [7] asserts that there exists a map ${\mathbb L}_{{\overline{\rho }}}: Z_n \rightarrow R_{{\overline{\rho }}}^{\Box }$ that is “compatible with local Langlands”, i.e. that for any map $x: R_{{\overline{\rho }}}^{\Box } \rightarrow \overline{{\mathcal K}}$, the composition $x \circ {\mathbb L}_{{\overline{\rho }}}$ is the map $Z_n \rightarrow \overline{{\mathcal K}}$ giving the action of $Z_n$ on $\Pi _x$, where $\Pi _x$ corresponds to $\rho _x$ under local Langlands. By Theorem 7.9 of [7], this conjecture implies Conjecture 1.3.1 of [5].

The paper [8] gives a further refinement of these conjectures. In particular, if one fixes ${\overline{\rho }}$ (or even merely the restriction ${\overline{\nu }}$ of ${\overline{\rho }}$ to prime-to-$\ell$ inertia) then this determines a primitive idempotent *e* of $Z_n$. This primitive idempotent is characterized by the following property: let $\Pi$ be an irreducible representation of $G_n$ over $\overline{{\mathcal K}}$, and let $\rho$ correspond to $\Pi$ via local Langlands. Let $\nu$ denote the unique lift of ${\overline{\nu }}$ to a representation over $\overline{{\mathcal K}}$. Then $\Pi$ lies in the block corresponding to *e* if, and only if, the restriction of $\rho$ to the prime-to-$\ell$ inertia group $I_F^{(\ell )}$ of *F* is isomorphic to $\nu$.

Section 9 of [8] constructs a finitely generated, reduced, $\ell$-torsion free *W*(*k*)-algebra $R_{\nu }$, a representation $\rho _{\nu }: W_F \rightarrow {\text {GL}}_n(R_{\nu })$, and an algebraic group $G_{\nu }$ with an action on ${\text {Spec}}R_{\nu }$ that are “universal” (in a sense made precise in [8], Proposition 9.2) for suitably rigidified representations of $W_F$ whose restriction to $I_F^{(\ell )}$ is isomorphic to $\nu$.

In section 10 of [8] we formulate two precise conjectures. First, we have the following conjecture, which we will call the “weak conjecture”:

### Conjecture 7.2

([8], Conjecture 10.2) There is a map ${\mathbb L}_{\nu }: eZ_n \rightarrow R_{\nu }$ “compatible with local Langlands” in the sense that for any $x: R_{\nu } \rightarrow \overline{{\mathcal K}}$, the composition with ${\mathbb L}_{\nu }$ is the map $eZ_n \rightarrow \overline{{\mathcal K}}$ giving the action of $eZ_n$ on the representation $\Pi _x$ corresponding to the specialization $\rho _{\nu ,x}$ of $\rho _{\nu }$ at *x* via local Langlands.

The map ${\mathbb L}_{\nu }$ is unique if it exists. Moreover, because the representation $\rho _{\nu }$ is $G_{\nu }$-invariant, the image of ${\mathbb L}_{\nu }$ is contained in the subring $R_{\nu }^{{\text {inv}}}$ of $G_{\nu }$-invariant elements of $R_{\nu }$. We then make the further “strong” conjecture:

### Conjecture 7.3

([8], Conjecture 10.3) The map ${\mathbb L}_{\nu }: eZ_n \rightarrow R_{\nu }$ of the “weak conjecture” identifies $eZ_n$ with $R_{\nu }^{{\text {inv}}}$.

The weak conjecture is easy to verify after inverting $\ell$. That is, Theorem 10.4 of [8] constructs a map:

$$\begin{aligned} {\mathbb L}_{\nu }\left[ \frac{1}{\ell }\right] : eZ_n \rightarrow R_{\nu }\left[ \frac{1}{\ell }\right] \end{aligned}$$

compatible with local Langlands; the weak conjecture amounts to showing that ${\mathbb L}_{\nu }[\frac{1}{\ell }](eZ_n)$ is contained in $R_{\nu }$. In fact, by Theorem 10.5 of [8], the image of $eZ_n$ under ${\mathbb L}_{\nu }[\frac{1}{\ell }]$ of $eZ_n$ is contained in the normalization ${\tilde{R}}_{\nu }$ of $R_{\nu }$. We denote by ${\tilde{\mathbb L}}_{\nu }$ the resulting map $eZ_n \rightarrow {\tilde{R}}_{\nu }$.

The goal of this section is to prove both of these conjectures for all *n*. The argument will be inductive, and makes use of the following key result, which is the main theorem of [8]:

### Theorem 7.4

([8], Theorem 11.1) Suppose that the “weak conjecture” holds for all $G_m$ with $m \le n$. Then the “strong conjecture” holds for all such $G_m$.

Both the “weak conjecture” and the “strong conjecture” hold for $G_1$ (this is an easy consequence of local class field theory). Thus to prove both the weak and the strong conjecture for $G_n$, it suffices to show:

### Theorem 7.5

Suppose the “strong conjecture” holds for $G_{n-1}$. Then the “weak conjecture” holds for $G_n$.

The main ingredients in the proof of this theorem are Theorem 5.1, together with a theory of $\gamma$-factors for families of representations of $W_F$. We first recall the latter theory, which was developed in [10].

If $\kappa$ is a field of characteristic zero containing *W*(*k*), and $\rho : W_F \rightarrow {\text {GL}}_n(\kappa )$ is a Weil group representation, then there is a rational function $\gamma (\rho , X, \psi )$ in $\kappa (X)$, called the Deligne–Langlands $\gamma$-factor of $\rho$. This $\gamma$-factor is compatible with the local Langlands correspondence, in the sense that if $\pi$ is an absolutely irreducible representation of $G_n$ over $\kappa$, and $\pi '$ is an absolutely irreducible representation of $G_{n-1}$ over $\kappa$, then the $\gamma$-factor $\gamma (\pi \times \pi ', X, \psi )$ of the pair $(\pi ,\pi ')$ coincides with the $\gamma$-factor $\gamma (\rho \otimes \rho ', X, \psi )$, where $\rho$ and $\rho '$ correspond to $\pi$ and $\pi '$ via local Langlands.

The main result of [10] extends this construction to families of Weil group representations. In particular, one has:

### Theorem 7.6

([10], Theorem 1.1) Let *R* be a Noetherian *W*(*k*)-algebra and let $\rho : W_F \rightarrow {\text {GL}}_n(R)$ be a representation that is $\ell$-adically continuous in the sense of [10, §2]. Then there exists an element $\gamma _R(\rho , X, \psi )$ of $S^{-1}R[X,X^{-1}]$ with the following properties:

1. If $f: R \rightarrow R'$ is a homomorphism of Noetherian *W*(*k*)-algebras, then one has:
  $$\begin{aligned} f(\gamma _R(\rho , X, \psi )) = \gamma _{R'}(\rho \otimes _R R', X, \psi ), \end{aligned}$$
  where we have extended *f* to a map $S^{-1}R[X,X^{-1}] \rightarrow (S')^{-1}R'[X,X^{-1}]$ in the obvious way.
2. If *R* is a field of characteristic zero, then $\gamma _R(\rho , X, \psi )$ coincides with the Deligne-Langlands gamma factor $\gamma (\rho ,X,\psi ).$

Note that if *R* is reduced and $\ell$-torsion free then the second property characterizes $\gamma _R(\rho ,X,\psi )$ uniquely; in particular it pins down the “universal $\gamma$-factors” $\gamma (\rho _{\nu },X,\psi )$ for all $\nu$. Since any $\rho$ arises by base change from some finite collection of these universal $\gamma$-factors, the two properties uniquely characterize the association $\rho \mapsto \gamma (\rho ,X,\psi )$.

### Proof of Theorem 7.5:

Suppose that Conjecture 7.3 holds for $G_{n-1}$, and fix a primitive idempotent *e* of $Z_n$, corresponding to a representation $\nu$ of $I_F^{(\ell )}$. We have a map: ${\tilde{\mathbb L}}_{\nu }: eZ_n \rightarrow {\tilde{R}}_{\nu }$. Let $Z'$ be the preimage of $R_{\nu }$ under the map ${\tilde{\mathbb L}}_{\nu }$; it suffices to show that $Z' = eZ_n$. Equivalently, it suffices to show that the map giving the action of $eZ_n$ on $eW_n$ factors through $Z'$ (as this map is the identity on $eZ_n$.) $\square$

On the other hand, since ${\tilde{R}}_{\nu }$ is finitely generated as an $R_{\nu }$-module, $eZ_n$ is finitely generated as a $Z'$-module. Thus, by Theorem 5.1, it suffices to show, for each primitive idempotent $e'$ of $Z_{n-1}$, that $\gamma (eW_n \times e'W_{n-1}, X^{-1}, \psi )$ and $\gamma (eW_n^{\iota } \times e'W_{n-1}, X, \psi )$ have coefficients in $Z' \otimes e'Z_{n-1}$.

Let $\nu '$ be the representation of $I_F^{(\ell )}$ corresponding to $e'$, and consider the map:

$$\begin{aligned} {\tilde{\mathbb L}}_{\nu } \otimes {\mathbb L}_{\nu '}: eZ_n \otimes e'Z_{n-1} \rightarrow {\tilde{R}}_{\nu } \otimes R_{\nu '}^{{\text {inv}}}. \end{aligned}$$

By Conjecture 7.3 for $G_{n-1}$, the map ${\mathbb L}_{\nu '}$ is an isomorphism of $e'Z_{n-1}$ with $R_{\nu '}^{{\text {inv}}}$, so $Z' \otimes e'Z_{n-1}$ is the preimage of $R_{\nu } \otimes R_{\nu '}^{{\text {inv}}}$ under ${\tilde{\mathbb L}}_{\nu } \otimes {\mathbb L}_{\nu '}$.

Since ${\tilde{\mathbb L}}_{\nu } \otimes {\mathbb L}_{\nu '}$ is compatible with the local Langlands correspondence, the image of $\gamma (eW_n \times e'W_{n-1}, X^{-1}, \psi )$ under ${\tilde{\mathbb L}}_{\nu } \otimes {\mathbb L}_{\nu '}$ is the gamma factor $\gamma _{R_{\nu } \otimes {R_{\nu '}}}(\rho _{\nu } \otimes \rho _{\nu '}, X^{-1},\psi )$. On the other hand, the intersection $(\tilde{R}_{\nu } \otimes R_{\nu '}^{{\text {inv}}}) \cap (R_{\nu } \otimes R_{\nu '})$ is equal to $R_{\nu } \otimes R_{\nu '}^{{\text {inv}}}$, because taking subrings of $G_{\nu '}$-invariants is compatible with flat base change. Thus $\gamma _{R_{\nu } \otimes R_{\nu '}}(\rho _{\nu } \otimes \rho _{\nu '}, X^{-1}, \psi )$ has coefficients in the tensor product $R_{\nu } \otimes R_{\nu '}^{{\text {inv}}}$. It follows that $\gamma (eW_n \times e'W_{n-1}, X^{-1},\psi )$ has coefficients in $Z' \otimes e'Z_{n-1}$ as claimed. The proof that $\gamma (eW_n^{\iota }\times e'W_{n-1},X,\psi )$ has coefficients in $Z'\otimes e'Z_{n-1}$ is similar. $\Box$

The inductive argument given above then gives:

### Corollary 7.7

Both Conjectures 7.2 and 7.3 hold for all *n*.

As an immediate consequence, we deduce that the elements of the Bernstein center constructed in [4], Proposition 3.11, are integral:

### Corollary 7.8

Let *w* be an element of $W_F$. Then there exists an element $z_w$ of $Z_n$ such that, for all irreducible smooth $\overline{{\mathbb Q}}_{\ell }$-representations $\pi$ of ${\text {GL}}_n$, the action of $z_w$ on $\pi$ is given by ${\text {tr}}\rho (w)$, where $\rho : W_F \rightarrow {\text {GL}}_n(\overline{{\mathbb Q}}_{\ell })$ corresponds to $\pi$ via local Langlands.

### Proof

For each $\nu$, consider ${\text {tr}}\rho _{\nu }(w)$. This is an element of $R_{\nu }^{{\text {inv}}}$, and hence corresponds to an element of $e_{\nu } Z_n$, which we denote by $z_{w,\nu }$. The desired element $z_w$ is the product, over all $\nu$, of the $z_{w,\nu }$.$\square$

## References

[1] Bernstein, J., Deligne, P.: Le “centre” de Bernstein, in Representations des groups reductifs sur un corps local. In: Deligne, P. (ed.) Travaux en cours. Hermann, Paris, pp. 1–32

[2] Bernstein J, Zelevinski A (1976). Representations of the group $$GL(n, F)$$ where $$F$$ is a nonarchimedean local field. Russ. Math. Surv..

[3] Bernstein J, Zelevinski A (1977). Induced representations of reductive $$p$$-adic groups, I. Ann. Sci. Ec. Norm. Sup..

[4] Chenevier, G.: Une application des variétiés de Hecke des groups unitaires, preprint (2011)

[5] Emerton, M., Helm, D.: The local Langlands correspondence for $$\text{GL}_n$$ in families. Ann. Sci. Éc. Norm. Supér. (4) 47(4), 655–722 (2014)

[6] Helm, D.: The Bernstein center of the category of smooth $$W(k)[\text{ GL }_n(F)]$$-modules. Forum of Math. Sigma vol. 4, e11 (2016). 10.1017/fms.2016.10

[7] Helm D (2016). Whittaker models and the integral Bernstein center for $$\text{ GL }_n$$. Duke. Math. J..

[8] Helm, D.: Curtis homomorphisms and the local Langlands correspondence in families, preprint arXiv:1605.00487 10.1007/s00222-018-0816-y PMC6413487 30956288

[9] Henniart G (1993). Caractérisation de la correspondance de Langlands locale par les facteurs $$\epsilon $$ de paires. Invent. Math..

[10] Helm, D., Moss, G.: Deligne–Langlands gamma factors in families, submitted arXiv:1510.08743v3 (2015)

[11] Moss G (2016). Gamma factors of pairs and a local converse theorem in families. Int. Math. Res. Not..

[12] Paškūnas Vytautas (2013). The image of Colmez’s Montreal functor. Publications mathématiques de l'IHÉS.

[13] Scholze P (2013). The local Langlands correspondence for $$\text{ GL }_n$$ over $$p$$-adic fields. Invent. Math..

